# *LRRK2* knockout mice have an intact dopaminergic system but display alterations in exploratory and motor co-ordination behaviors

**DOI:** 10.1186/1750-1326-7-25

**Published:** 2012-05-30

**Authors:** Kelly M Hinkle, Mei Yue, Bahareh Behrouz, Justus C Dächsel, Sarah J Lincoln, Erin E Bowles, Joel E Beevers, Brittany Dugger, Beate Winner, Iryna Prots, Caroline B Kent, Kenya Nishioka, Wen-Lang Lin, Dennis W Dickson, Christopher J Janus, Matthew J Farrer, Heather L Melrose

**Affiliations:** 1 Department of Neuroscience, Mayo Clinic, Jacksonville, Florida, 32224, USA; 2 Junior Group III, Interdisciplinary Center for Clinical Research, Nikolaus-Fiebiger Center for Molecular Medicine, FAU, Erlangen-Nürnberg, Germany; 3 Department of Neuroscience, Center for Translational Research in Neurodegenerative Disease, University of Florida, Gainesville, Florida, 32610, USA; 4 Department of Medical Genetics, University of British Columbia, Vancouver, V6T 285, Canada

**Keywords:** Parkinson’s disease, Knockout, Dopamine, Microdialysis, Neuropathology, Open-field, Motor co-ordination, Kidney, Autophagy

## Abstract

Mutations in the *LRRK2* gene are the most common cause of genetic Parkinson’s disease. Although the mechanisms behind the pathogenic effects of *LRRK2* mutations are still not clear, data emerging from *in vitro* and *in vivo* models suggests roles in regulating neuronal polarity, neurotransmission, membrane and cytoskeletal dynamics and protein degradation.

We created mice lacking exon 41 that encodes the activation hinge of the kinase domain of LRRK2. We have performed a comprehensive analysis of these mice up to 20 months of age, including evaluation of dopamine storage, release, uptake and synthesis, behavioral testing, dendritic spine and proliferation/neurogenesis analysis.

Our results show that the dopaminergic system was not functionally comprised in *LRRK2* knockout mice. However, *LRRK2* knockout mice displayed abnormal exploratory activity in the open-field test. Moreover, *LRRK2* knockout mice stayed longer than their wild type littermates on the accelerated rod during rotarod testing. Finally, we confirm that loss of LRRK2 caused degeneration in the kidney, accompanied by a progressive enhancement of autophagic activity and accumulation of autofluorescent material, but without evidence of biphasic changes.

## Background

Mutations in the *LRRK2* gene, originally described in 2004, have now emerged as the most important genetic finding in Parkinson’s disease (PD) [1,2]. Incredibly, the most common mutation LRRK2 G2019S accounts for up to 40 % of Parkinsonism in populations of certain ethnic descent [3-5]. *LRRK2* mutations also account for around 2% of sporadic Parkinsonism and two risk factors have been identified in Asian populations [6-9]. *LRRK2*-associated PD is a late onset disease and in general the disease resembles idiopathic PD both clinically and pathologically.

LRRK2 has been linked to neurite outgrowth, vesicular trafficking, protein translation and autophagy [10]. Analysis of mutant transgenic and knock-in models expressing physiological levels of LRRK2 has led to an emerging theme that aberrant LRRK2 leads to subtle alterations in dopamine neurotransmission, albeit in the absence of dopaminergic neuronal loss [11]. Imaging studies in asymptomatic PD patients show the earliest detectable changes occur in the dopamine transporter and the same holds true for asymptomatic *LRRK2*[12,13] and *SNCA* (*alpha-synuclein*) patients [14-16]. Neurotransmission alterations in mutant LRRK2 models may be similar to early preclinical events, suggesting an early involvement in dopamine dysfunction.

The expression profile of *LRRK2* mRNA suggests that LRRK2 is unlikely to be an essential developmental protein [17]. In adult rodent brain, *LRRK2* mRNA is found at highest levels in dopamine receptive areas particularly the striatum [18-22]. However, protein expression of LRRK2 is abundant throughout the brain including the substantia nigra, striatum, hippocampus, thalamus, cerebellum and cortex [23] suggesting it may have a role in multiple brain functions (i.e. memory, sensory, emotion) and not just those involved in motor control.

To further study the role of LRRK2 in the brain, we have developed *LRRK2* knockout mice by ablating exon 41 in the kinase domain of LRRK2. We have performed a comprehensive analysis to study the effect of loss of LRRK2; this includes a thorough investigation of the dopaminergic system, extensive behavioral tests to examine motor, co-ordination and emotional behavior, as well as neuropathological analyses. We find little evidence that loss of LRRK2 impacts dopaminergic neurotransmission or striatal behaviors, however we present data showing changes in the exploratory and motor co-ordination behaviors in these mice. These findings may be an important consideration for future anti-LRRK2 therapies.

## Results

### Generation of murine *LRRK2* knockout mice

**Figure 1:**
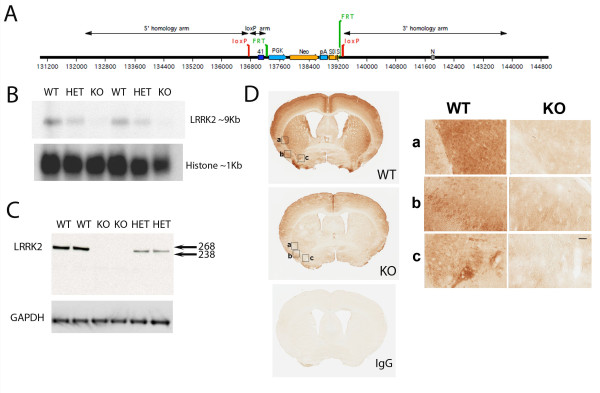
**Generation and expression characterization of*LRRK2*KO mice.(a)** Schematic diagram (courtesy of Ozgene PLC) showing targeted locus. Exon 41 was flanked with LoxP sites to allow deletion with Cre recombinase. PKG-Neo-pA-SD-IS is Ozgene’s standard selection cassette and was inserted downstream of exon 41. The PKG-neo cassette was also flanked by FRT sites to allow FLPe recombinase deletion. The targeting vector was constructed from three fragments, the 5’homology arm, the 3’ homology arm and the lox P arm, which were generated by PCR. Splicing of exon 40 to 42 causes a frame shift mutation, with the introduction of an early stop codon (TGA). **(b)** Northern blot hybridized with a probe to *LRRK2* exon 24–27 showing absence of transcript in KO and diminished transcript in HET. A *histone* probe was used as loading control. **(c)** Immunoblot with LRRK2 antibody 1182E, raised to amino acids 841–960 showing the absence of LRRK2 protein in KO and diminished signal in HET. GAPDH was used as a loading control. **(d)** Immunohistochemistry with MJFF2 c41-2 antibody showing WT and KO brain sections at the level of the striatum. Specific signal is seen in the WT compared to KO. Rabbit IgG was used as an isotype control. Boxes depict enlarged images to the right. Scale bar 50 microns.

The targeting strategy for generation of *LRRK2* knockout (KO) mice is shown in Figure 1A. Homozygous mice received from Ozgene PLC were bred to Jackson C57BL6/J mice and subsequent heterozygous offspring were bred together to obtain wild type (WT), heterozygous (HET) and KO animals. Northern blotting analysis with a probe designed to *LRRK2* exon 24–27 confirmed the absence of the ~9 kb LRRK2 mRNA transcript in KO mice and a reduced transcript signal in the HET mice (Figure 1B). Similarly, immunoblotting confirmed absence of LRRK2 protein band in the KO mice and a diminished signal in HET mice (Figure 1C). Immunohistochemistry also revealed specific signal in the WT compared to KO (Figure 1D).

To determine if any compensatory mRNA changes occurred in the paralog *LRRK1*, we used quantitative Taqman assay with a probe to murine *LRRK1* to compare WT, HET and KO mice. No significant alterations in *LRRK1* mRNA levels were observed in any of the brain regions tested. We also checked mRNA levels of *SNCA*, *MAPT* or *PARKIN* and found no differences ( Additional file 1: Figure S1).

*LRRK2* KO mice were fertile and appeared to be healthy from birth, with body weights comparable to WT littermates within the study period. HET x HET breedings yielded Mendelian ratios in line with expected inheritance (from over 70 litters 24.4 % WT, 51.6 % HET, 24 % KO).

Subsequent characterization experiments were performed to compare KO and WT mice at different aging time points. Due to animal costs and space restraints, we included a HET group in some, but not all, of these analyses.

### Dopaminergic system characterization

**Figure 2:**
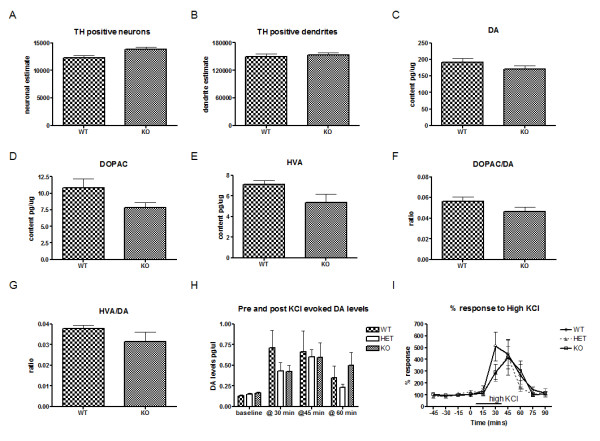
**Dopaminergic characterization in*LRRK2*KO mice. (a,b)** Unbiased sterological estimates for TH-positive **(a)** neurons and **(b)** dendrites in the substantia nigra reveal no difference between WT and *LRRK2* KO mice. Counting was performed using optical fractionator probes in TH stained sections from 18 month old WT and KO mice. **(c-e)** Dopamine axon terminal neurochemistry is normal in *LRRK2* KO mice. HPLC analysis of total striatal content of dopamine **(da)** and its metabolites 3,4-dihydroxyphenylacetic acid (DOPAC) and homovanillic acid (HVA). **(f-g)** Mean of individual animal turnover ratios of DA to metabolites DOPAC and HVA. **(h-i)***In vivo* microdialysis reveals similar baseline and KCl-evoked levels of extracellular DA in WT, HET and KO mice **(i)**. DA levels were plotted as a percentage of each individual animal’s mean baseline levels to compare response over time **(h)** % Response did not differ between WT, HET and KO mice. Dopamine levels were measured by HPLC from dialysates collected from the striatum. All data are presented as mean ± SEM.

*LRRK2* expression is normally found in high levels in the striatum so we reasoned that loss of *LRRK2* may impact the functional integrity of the nigro-striatal dopaminergic pathway. To determine if dopamine neurons or their processes were altered in the substantia nigra, we performed stereological counts of tyrosine hydroxylase (TH) positive neurons and dendrites in KO and WT mice aged 18–20 months. No differences in neuronal estimates were observed between KO mice (mean estimate ± SEM; 13,766 ± 471) compared to their WT littermates (12,267 ± 481) (Figure 2A). Similarly, there were no differences between dendritic estimates for WT (149,763 ± 6213) and KO (153,873 ± 4351) (Figure 2B). Next, we examined dopamine axonal neurochemistry by analyzing total dopamine content in striatal lysates by HPLC from 10 month and 18 month KO mice and WT littermates (Figure 2C-G, data shown only for 18 months) and found dopamine and its metabolite 3,4-dihydroxyphenylacetic acid (DOPAC), and homovanillic acid (HVA) levels were equivalent in KO and WT animals at both time points.

Since total striatal dopamine includes dopamine that is stored, synthesized, released and taken up, we hypothesized that we may observe differences in KO mice by investigating these mechanisms independently. First, we performed microdialysis to measure extracellular release of dopamine *in vivo*. Extracellular dopamine levels were measured before and after KCl-evoked dopamine response in WT, HET and KO mice aged 3–4 months of age (Figure 2H). Baseline levels of dopamine were not found to differ between WT (0.13 ± 0.06 pg/μl), HET (0.15 ± 0.06 pg/μl) or KO mice (0.16 ± 0.07 pg/μl). When post-KCl dopamine levels were normalized to the average of their individual basal dopamine levels (i.e. % response) no significant differences were observed (Figure 2I). Next, we examined dopamine uptake in the WT, HET and KO mice using a radioactive uptake assay with [3H] dopamine. Uptake was comparable across the three groups suggesting a functionally intact dopamine transporter ( Additional file 2: Figure S2A). Concluding that release, storage and uptake of dopamine appear to be normal, we then examined D2-autoreceptor mediated synthesis and release, which is one of the feedback mechanisms in the striatum. It is well known that antagonizing pre-synaptic D2 autoreceptors can remove this feedback inhibition and cause increased synthesis as well as release of dopamine [24]. WT, HET and KO mice were treated with the D2 receptor antagonist raclopride, sacrificed 30 min later and HPLC analysis performed to quantify dopamine and metabolites. As expected, dopamine turnover, as measured by the ratio of dopamine metabolites to stores of dopamine increased dramatically [One way ANOVA *ps* < 0.0001 for DOPAC/dopamine and HVA/dopamine] following treatment with raclopride ( Additional file 2: Figure S2B, C). However, Tukey’s post hoc comparisons did not reveal differences in this response between WT, HET or KO mice, suggesting that autoreceptor-mediated feedback is normal in the absence of LRRK2. A significant increase in levels of serine-40 phosphorylated TH after raclopride treatment was also observed (indicating increased synthesis), but this response was not different between WT, HET or KO mice (data not shown). Finally, we examined striatal receptor density for both dopamine D1 and D2 receptors by autoradiography in 10 and 18 month old WT and KO mice and no differences were observed ( Additional file 3: Figure S3A-D).

#### *Analysis of striatal dendritic spines*

We have previously shown that LRRK2 G2019S transgenic mice have impaired neurite outgrowth *in vitro*[25] and *in vivo*[26] as well as a reduced number of mature spines in the hippocampus [26]. We have also reported that neurite outgrowth was increased in both midbrain and hippocampal cultures derived from *LRRK2* KO mice [25]. As LRRK2 has previously been linked to ERM proteins and the actin cytoskeleton [27-31] we hypothesized that the loss of LRRK2 in the striatum in KO mice, an area where it is normally highly expressed, may impact on striatal dendritic spines. We counted spine number in Golgi-Cox labeled medium spiny neurons (MSN) of brains from aged (18+ months) KO and WT mice. Spines were classified into different morphological types [32]. No differences were found in spine numbers between in KO and WT mice suggesting that lack of LRRK2 has no effect on MSN spine dynamics *in vivo* ( Additional file 4: Figure S4).

### Proliferation / Neurogenesis studies

As LRRK2 is normally expressed in the hippocampal dentate gyrus and proliferation, neurogenesis and migration are impaired in G2019S BAC mice [26], we sought to ascertain whether loss of expression has an impact on cell proliferation. We analyzed the effect of loss of LRRK2 on newly generated cells in the hippocampal dentate gyrus after a single injection of BrdU. Unbiased stereological counting methods were applied to estimate the number of BrdU-labeled cells. No differences were observed in proliferation between KO and WT mice ( Additional file 5: Figure S5A). Since doublecortin (DCX) expression levels in adult brain reflect neurogenesis [33], we also performed counts on DCX labeled neurons in the hippocampus and these were also found to be comparable in KO and WT mice ( Additional file 5: Figure S5B) suggesting normal neurogenesis occurs in KO mice.

### Neuropathology

Hameotoxylin and eosin revealed morphology of KO mouse brains to be normal up to 20 months of age. To assess mice for neuropathological alterations we examined young (3–6 months), middle aged (10–13 months) and aged (18+ months) WT, HET and KO mice for a variety of markers associated with Parkinson’s disease pathology including alpha-synuclein, tau and Iba-1. In agreement with a previous report [34], no overt differences were observed with any of these markers between WT, HET and KO mice. As we have previously shown alterations in tau regulation in our BAC mutant LRRK2 G2019S model, we performed quantitative immunoblotting with tau antibodies (tau-5, CP-13 and tau-1) in lysates from aged WT and KO mice. Our results indicate that tau regulation is normal in KO mice ( Additional file 6: Figure S6).

### Behavioral Analysis

#### Open-field Test

In the initial characterization, we focused on the analysis of spontaneous exploratory locomotor behavior evaluated in the open-field test. The open-field test assesses spontaneous exploration of mice as well as their emotional response after being exposed to much larger than a home cage and brightly illuminated unfamiliar environment [35-37]. The rationale behind this approach was based on evidence that anxiety disorders, which affect about 40 % of PD patients [38,39] and other psychiatric symptoms can precede the onset of motor symptoms, such as bradykinesia, rigidity, resting tremor and postural instability in PD by decades [39]. The open-field test, which combines the evaluation of anxiety as well as spontaneous locomotor activity, therefore presents an appropriate paradigm for the phenotyping characterization of the KO mice. The test has been successfully used with other familial Parkinson disease mouse models, for example mice deficient in parkin gene [40].

We characterized the behavior of KO and WT mice in the open-field test, adopting longitudinal experimental design, at a young (7 months) and older (16 months) time point. The evaluation was done in one 5-min session at each age in order to minimize the saving effect and habituation to the test due to repeated testing. Data was analyzed by repeated measures ANOVA followed by Student’s t-tests for independent samples (to assess the genotype effect at each testing age) and matched-pairs samples (to assess the age effect within each genotype).

**Figure 3:**
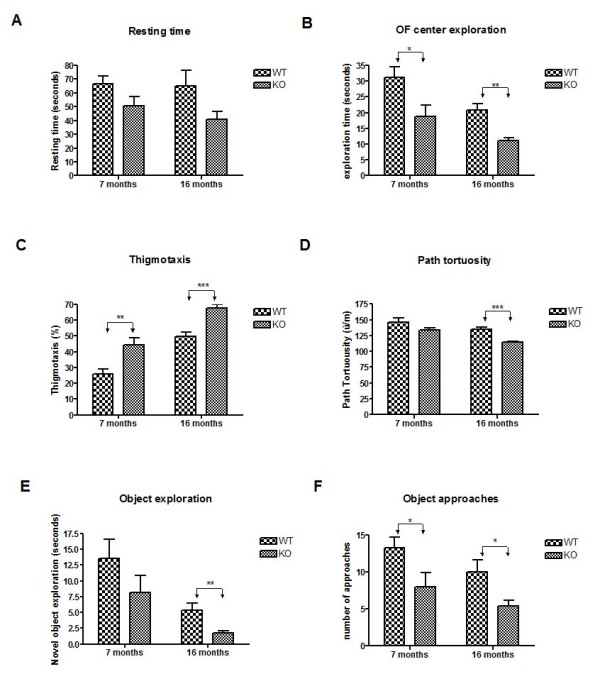
**Open-field test reveals abnormal exploratory behavior in*LRRK2*KO mice at both 7 and 16 months of age.(a)** KO mice tended to spend longer time on rest stops longer than 5 seconds. **(b)** KO mice spent significantly less time exploring the inner area of the open-field at both testing ages. **(c)** KO mice displayed significantly greater thigmotaxic (wall hugging) behavior at both testing ages. **(d)** KO mice take less turns and their travel path is less curvy than WT mice, this was significant at 16 months. **(e)** Aged KO mice spend less time exploring the object than their WT counterparts. **(f)** KO mice of both age time points approach the object less frequently. Data presented as mean ± SEM. Data was analyzed by repeated measures ANOVA (see results section for full details) followed by Student’s t-tests for independent samples (to assess the genotype effect at each testing age) and matched-pairs samples (to assess the age effect within each genotype). The p-values on the graphs reflect the independent sample t-test results. ** p <0.01, ***p <0.001.

Overall analysis of the exploration of the open-field arena by KO and WT mice revealed no differences in the length of walking distance, walking speed or onset of exploration between the genotypes. The KO mice tended to spend more time on longer (> 5 s) rests than WT mice during their exploration (p < 0.05, ANOVA, genotype effect), however, high variability of this behavior did not yield significant differences at α = 0.05 at each age of testing (Figure 3A). The number of rests and shorter (< 5 s) stops did not differ between the genotypes.

Mice of both genotypes explored the center of the arena less at 16 months than at 7 months test (15.9 s ± 1.6 for 16-month and 25.0 s ± 3.5 for 7-month mice respectively, p < 0.001, ANOVA, age effect). However, KO mice explored the center of the open-field arena significantly less than the WT mice (15.0 % ± 2.4 and 25.9 % ± 2.8 of time averaged across age for KO and WT mice respectively, p < 0.01, ANOVA, genotype effect). The difference was significant at both at 7 (p < 0.05, t-test) and 16 (p < 0.01, t-test) months of age (Figure 3B).

**Figure 4:**
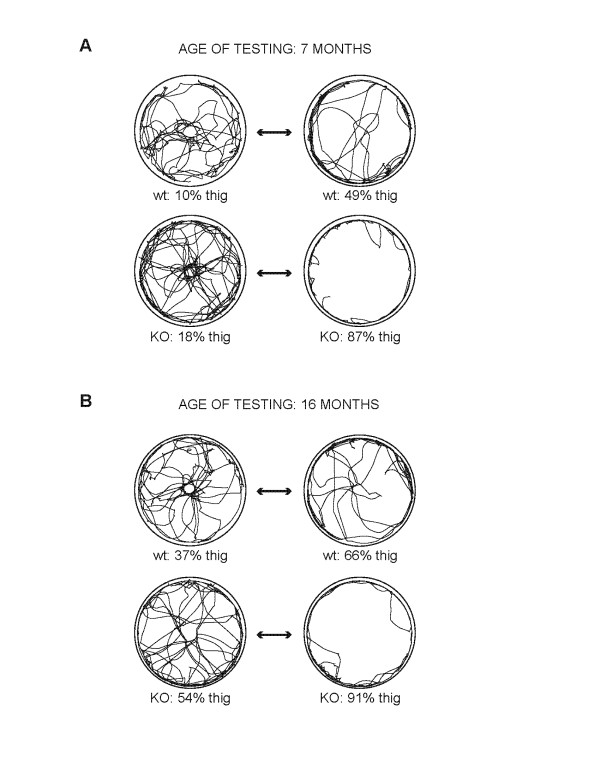
***LRRK2*KO mice exhibit thigmotaxic behavior in the open-field test.** Examples of movement traces from open-field trials show the range of thigmotaxic behavior from **(a)** 7 months and **(b)** 16 months old WT and KO mice. This thigmotaxic behavior was typical of *LRRK2* KO mice, although a few displayed normal exploration patterns.

This shorter time of exploration of the inner area of the field by the KO mice was likely due to their increased thigmotaxic (wall hugging) behavior (p < 0.001, ANOVA, genotype effect). The KO mice spent between 18 % to 87 % and 53 % to 91 % of their exploratory activity moving within close proximity of the wall during 7- and 16-month tests respectively. In contrast, the WT controls showed thigmotaxic behavior within a range of 9 % to 48 % and 36 % to 66 % during respective tests. Representative examples of the exploratory paths, reflecting the differences in the thigmotaxic behavior, are presented in Figure 4. Overall, the thigmotaxic behavior increased with age (p < 0.001, ANOVA age effect), with no significant interaction between the genotype and age factors. The differences in thigmotaxic behavior between the genotypes were significant at both 7 (p < 0.01, t-test) and at 16 (p < 0.001, t-test) months of age (Figure 3C).

Our analysis also revealed that overall, the exploratory paths of KO mice were less curvy and tortuous as compared to the paths of the WT counterparts (p < 0.001, ANOVA, genotype effect) and path tortousity was lower in mice of both genotypes during tests at 16 months (p < 0.001, ANOVA, age effect). The specific comparisons revealed a significant genotype effect on path tortuosity only at 16 months (p < 0.001, t-test, Figure 3D).

Lastly, the KO mice approached an object placed in the center of the arena with longer latencies than WT mice (p < 0.05, ANOVA, genotype effect). In general, KO mice tended to spend less time exploring the object than WT mice (averaged across age 4.96 ± 0.09 and 9.45 ± 0.15 seconds respectively, p = 0.06, ANOVA, genotype effect). The borderline significance of the genotype effect was due to high variability in this behavior at 7-months (Figure 3E). At 16 months, however, the KO mice explored the object significantly less (p <0.05, t-test, Figure 3E). The KO mice also approached the object less frequently (p < 0.05, ANOVA, genotype effect), with significant differences at each age of testing (p*s* < 0.05, t-test for 7 and 16 months, Figure 3F). All mice approached the object less frequently when tested at the older age (p < 0.05, ANOVA, age effect).

In summary, our results indicate that the KO mice showed abnormal spontaneous exploratory behavior in the open-field test, which was characterized by significantly higher thigmotaxic exploration of the arena. This result might be indicative of increased anxiety in the KO mice. Thigmotaxic behavior has been previously reported as a reliable measure of anxiety in the pre-clinical studies testing anxiolytic drugs [41]. In spite of the observed differences, we interpret these results cautiously, since most of the open-field test measures are interdependent [37]. We focused our emphasis on thigmotaxic behavior since it is likely that the initial prevalent engagement in this behavior affects other measures, like the exploration of inner area (r^2^ = 0.49, and r^2^ = 0.72 for 7 and 16 months respectively, p*s* = 0.001), and path tortuosity (r^2^ = 0.38 – 7 months, and r^2^ = 0.45 – 16 months, p*s* < 0.001).

### Motor tests

**Figure 5:**
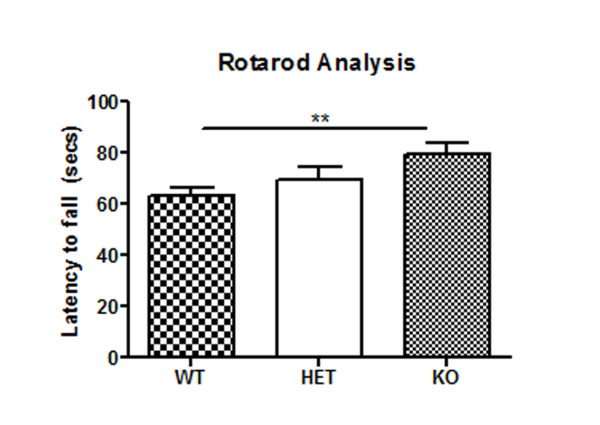
**Alterations in motor co-ordination behavior in*LRRK2*KO mice.** Motor co-ordination and balance was tested by accelerating rotarod analysis in a 7-month cohort of WT, HET and KO mice. The speed of the rod was set to 4-40 rpm acceleration, increasing 1 rpm every 5 seconds. KO mice significantly outperformed their WT littermates. Data are plotted as mean ± SEM and were analyzed by ANOVA with Tukey’s post-hoc comparisons. ** p <0.01.

Next, in a separate 7 month old cohort, we examined motor behavior in KO mice and compared this to both WT and HET littermates. Motor co-ordination/balance was examined by rotarod performance and gait was examined by digigait footprint analysis. Since the weights of the mice in each genotype did not differ significantly (p > 0.2, ANOVA), body weight was not used as a covariate in the analysis [42]. Analysis of performance on an accelerating rod revealed significant differences between the groups (p < 0.02, ANOVA). Subsequent, post-hoc analysis revealed that KO mice performed significantly better than their WT littermates, spending ~20 % (p < 0.01, Tukey’s test) longer on the rod (Figure 5).

The gait analysis was performed at three different speeds (14, 18 and 24 cm/sec) and compared a number of spatial and temporal indices between WT, HET and KO mice. Overall, there were no significant differences between the genotypes for stride lengths or any other of the recorded indices (Table 1) suggesting gait and thus striatal function remains intact.

**Table 1:** **Normal gait in*LRRK2*KO mice**. Mouse gait dynamics were obtained using a motorized treadmill by ventral plane videography and analyzed with DigiGait® software

**Average gait dynamics in WT, HET & KO mice**				
**treading at a speed of 24cm/sec**				
**Parameter**	**WT (n=9)**	**HET (n=8)**	**KO (n=8)**	**p**
Stride length (cm)	6.42 ± 0.57	6.38 ± 0.71	6.09 ± 0.50	ns
Stride frequency (steps/sec)	3.84 ± 0.37	3.88 ± 0.46	4.06 ± 0.32	ns
Stride duration (sec)	0.267 ± 0.023	0.265 ± 0.029	0.254 ± 0.020	ns
% Stance duration	58.5% ± 3.3%	59.5% ± 5.5%	59.1% ± 4.4%	ns
% Swing duration	41.5% ± 6.2%	40.5% ± 6.0%	41% ± 3.9%	ns
Step angle (deg)	61.1 ± 9.6	64.3 ± 6.5	59.1 ± 6.8	ns
Fore paw angle (deg)	9.63 ± 1.68	11.76 ± 5.04	12.51 ± 4.13	ns
Hind paw angle (deg)	18.97 ± 3.13	20.08 ± 1.81	18.43 ± 2.42	ns
Total steps (No./paw)	17 ± 5	18 ± 4	18 ± 4	ns
Hind limb shared stance (sec)	0.047 ± 0.026	0.050 ± 0.018	0.036 ± 0.013	ns

Values are means ± StdDev and were analyzed by one-way ANOVA; ns = p>0.05.

Gate indices were not different between WT, HET and KO mice. Data are plotted as mean ± SD and were analyzed by ANOVA.

### Inflammatory and degenerative alterations in kidney of *LRRK2* KO mice

**Figure 6:**
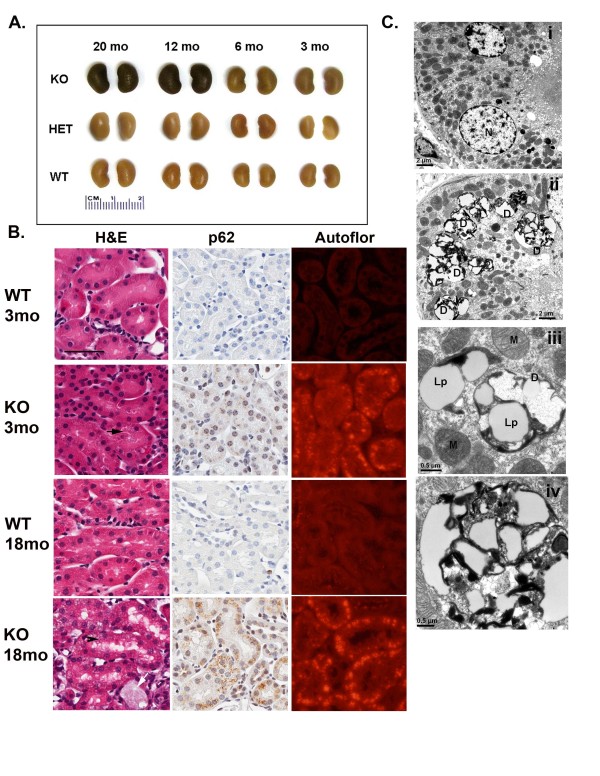
***LRRK2*KO mice develop degeneration in the kidney from an early age.(a)** Comparison of perfused kidneys extracted from WT, HET and KO mice at 3, 6, 12 and 20 months of age. Coloration changes were observed as early as 3 months in KO mice. HET kidneys were not affected. **(b)** Histopathology of kidney reveals morphological changes. H&E at 3 months shows pigmentation in proximal tubule (arrow). H&E at 18 months shows extensive vacuolization (white arrow) and pigmentation (arrowhead). Positive p62 staining can be seen at 3 months in KO and is extensive by 18 months. Pigmentation in tubules, most likely to be lipofuscin, is autofluorescent in rhodamine channel at both 3 and 18 months in KO. Scale bar is 50 microns. **(c)** Electron microscopy in KO kidney reveals degenerative changes at the ultrastructural level in renal cortex tubular epithelial cells from 18 month old KO mouse. (i) Normal epithelial cell in WT mouse. (N) nucleus. (ii) Degenerating debris **(d)** in tubular epithelial cell in KO. (iii) Higher power image from KO showing normal mitochondria (M), lipid (Lp) probably lipofuscin and degenerative structure, probably lysosomal-containing lipid. (iv) Higher power image from KO of degenerative structure showing extensive debris.

**Figure 7:**
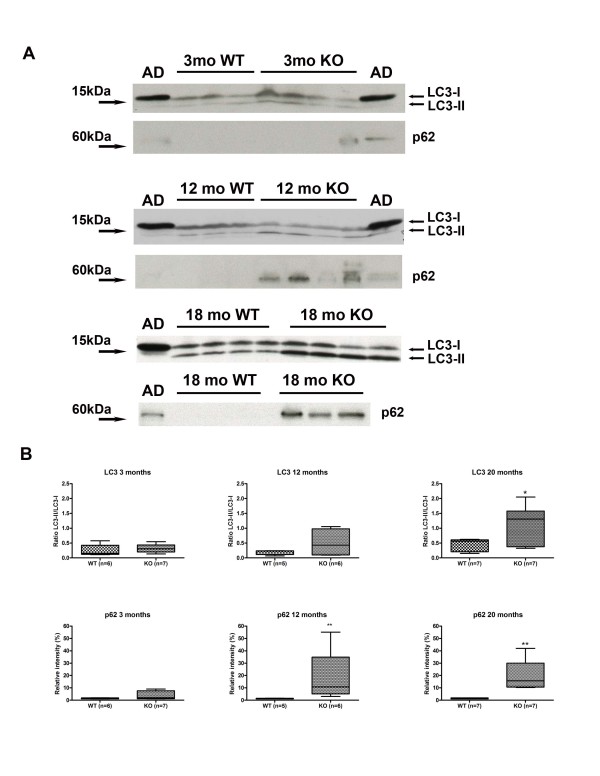
**Quantitative differences in p62 levels and LC3-II/LC3-I ratio in aged*LRRK2*KO mice.(a)** Representative LC3 and p62 immunoblots of insoluble fraction kidney lysates from 3-, 12- and 20-month old WT and KO mice. Visibly more p62 is seen in both 12- and 20-month old mice. LC3-II levels are also visibly increased in 20-month old KO mice. **(b)** Graphical presentation of densitometric quantification of immunoblots from N = 5-7 animals for each group. Alzheimer (AD) brain lysate was used as a control. LC3 data are expressed as a ratio of LC3-II/LC3-I. The ratio of LC3-II/LC3-I was significantly increased by 20 months, indicating an increase in autophagy. p62 data was expressed as % intensity, normalized to the lowest densitometric band on each blot. A significant increase was observed in KO mice at 12 and 20 months. Data are presented as mean ± SEM and were analyzed by either Student’s t-test or Mann Whitney non-parametric comparisons. ** p <0.01, ***p <0.001.

Two groups have previously identified abnormalities in the kidneys of *LRRK2* KO mice including morphological changes, lysosomal and autophagic alterations, vacuolization and accumulation of pigment [34,43]. We examined kidneys from mice aged 3 to 20 months and also observed coloration (darkening) changes beginning as early as 3 months (Figure 6A). Significant enlargement was observed in the kidneys from the oldest mice (mean ± SEM; for KO female 0.23 ± 0.01 g and female WT 0.19 ± 0.01 g, p < 0.05 t-test; male KO 0.35 ± 0.02 g and male WT 0.22 ± 0.01 g, p < 0.001 t-test). At the gross level, kidneys from HET mice did not differ from WT counterparts (Figure 6A) therefore we limited subsequent analysis to KO and WT. Hematoxylin and eosin staining revealed inflammatory abnormalities, vacuolization and pigmentation in KO kidneys as young as 3 months, which was progressive with age (Figure 6B). We performed histological staining for a number of different markers including p62 (an ubiquitin-binding protein involved in cell signaling, oxidative stress and autophagy, found to be upregulated in many neurodegenerative diseases), Periodic acid-Schiff (PAS) stain for glycoproteins, iron (Gomori's Prussian blue) and melanin (Fontana-Masson) staining for pigment and immunohistochemistry for PD related proteins alpha-synuclein and ubiquitin. The KO kidney pigment was negative for iron and melanin. PAS staining revealed abnormal granular staining reminiscent of lipofuscin in the proximal tubules of KO mice as young as 3 months of age, progressing at 12 months and becoming severe by 18 months (data not shown). These granular inclusions were also autofluorescent, the intensity increasing with age (Figure 6B). Increased p62 immunostaining in the same cells in KO mice was observed as early as 3 months of age, although quantitative differences were not detectable by immunoblot at this age (Figure 7). The intensity of p62 immunostaining progressed with age, and by 12 months quantitative differences were evident. By 18 months the KO mice had on average a 15-fold increase (p < 0.01, t-test) in immunoblot relative band density compared to WT controls (Figure 7). We were unable to corroborate the previous reports of increases in alpha-synuclein or ubiquitin [34,44] in KO mice neither by immunohistochemistry nor by immunoblotting.

To further examine the granular pathology at the ultra-structural level, we performed electron microscopy in kidneys from 18 months old KO and WT mice (Figure 6C). Tubular epithelial cells in the renal cortex of KO kidney were found to contain degenerating debris and accumulation of pigment resembling lipofuscin, both absent from WT kidney. Although the debris was significant, some normal tubular epithelial cells were still present and no evidence of abnormal mitochondria was found. Following on from conflicting reports of impaired/biphasic or no change autophagy in *LRRK2* KO mice [34,43,44] we performed immunoblotting with LC-3 antibody in kidney lysates from mice aged 3, 12 and 18 months old. No significant differences were seen at the 3 and 12 month time-point. However in KO mice aged 18 months we observed a significant 2.8 fold increase in intensity of the LC-3 II band (p < 0.05, t-test) indicating an increase, rather than the previously reported decrease.

## Discussion

In this report we present dopaminergic, behavioral and pathogenic characterization of mice lacking LRRK2 to 20 months of age. Our most significant findings are observed at the behavioral level, which reveal normal motor gait but altered open field and motor co-ordination behavior in *LRRK2* KO mice. In the open-field test KO mice displayed an increased thigmotaxic behavior, walking along the wall of the apparatus, which resulted in reduced path tortuousity. This phenotyping profile was characteristic both at 7 and 16 month of age, with no indication of progressing deterioration in this phenotype. It has been reported that the measures obtained in the open-field test are variable and labile [45], which could account for the lack of significant interactions between age and genotype in our study. Increased thigmotaxic behavior is often attributed to an increase in anxiety during the exploration of new environment [35,41] or lack of flexibility in changing ongoing behavior [46]. Follow up studies to examine progression of these behaviors in KO mice will require larger cohort sizes and will focus on complementary to the open-field tests evaluating anxiety in mice, including rescue with anxiolytic agents. Interestingly, we have previously reported very similar open field behaviors in our mutant G2019S BAC mice [47] which formulates the idea this phenotype could be a loss of function behavior. While current thinking favors a gain of function role for aberrant LRRK2, the phenotype reported in *LRRK2* KO kidneys [34,43] has somewhat challenged this, leading to speculation of cell-specific LRRK2 roles.

Surprisingly, our study revealed that *LRRK2* KO mice stayed persistently longer on the rotating rod, despite no obvious differences in their gait characteristics. Coincidentally, the Michael J Fox foundation recently posted online data detailing phenotypic testing of their *LRRK2* KO model being characterized at Wil Research and this data showed a trend for enhanced rotarod performance in 4 month old KO mice compared with WT controls http://www.pdonlineresearch.org/sites/default/files/MJFF%20Animal%20Models%20Data%20-%20Oct%2031.pdf). The rotarod test is known to be sensitive to cerebellar function and deficits in cerebellar purkinje neurons generally result in a reduced rotarod performance. In the literature there are only a few reports of genetic mouse models exhibiting enhanced rotarod performance compared with their wild type/ non-transgenic littermates. Examples include a mouse model for Down’s syndrome (Ts65DN) [48], heregulin (a ligand for tyrosine receptor kinase) mutant mice [49], an epilepsy model deficient in a repair protein L-isoaspartate (d-aspartate)-*O*-methyltransferase (*Pcmt1−/−*) [50] and Huntington triplet deletion mice *Hdh* (∆Q/∆Q) mice [51]. Both the Ts65DN and heregulin model had known cerebellar morphological changes [48,49]. Morphological and pathological analysis of the cerebellum in our *LRRK2* KO and HET mice did not reveal any obvious structural differences, however given the high expression of LRRK2 in the cerebellum, further studies examining cerebellar neurochemistry/function may be warranted. In the heregulin mutant and *Pcmt1*−/− models, enhanced rod performance was also accompanied by hyperactivity [49,50] which we did not observe. However, like our *LRRK2* KO mice, the *Pcmt1*−/− mice also displayed significant thigmotaxic behaviors in addition to enhanced rotarod phenotype. Our rotarod result together with the results obtained in the open-field test might indicate inability of termination of ongoing behavior, which resulted in higher ceiling performance of KO mice in the rotarod test.

It is also important to note that aside from the cerebellum, enhanced rod performance could also be attributed to central (i.e. heart) and/or peripheral effects (i.e. muscle). Since LRRK2 is expressed in both heart and skeletal muscle [17] a closer physiological examination of heart and muscle may also be revealing.

*LRRK2* KO mice have normal lifespans and do not have any compensatory changes in *LRRK1* or other PD related mRNAs. In agreement with previous reports, and consistent with the lack of striatal-related motor phenotypes, we did not observe any changes in total striatal dopamine levels nor did we observe nigral neuronal loss. Given that several mutant *LRRK2* models have normal total dopamine levels, but still exhibit subtle defects in extracellular release, we extended on the studies of others [34,43] and performed *in vivo* microdialysis in *LRRK2* KO, HET and WT mice. Endogenous extracellular levels of dopamine were found to be normal in KO and HET mice compared with WT, as were post-KCl stimulation levels. Taken together, our data suggest that the dopamine system is functionally intact in LRRK2 KO mice. Future studies to examine extracellular release of other neurotransmitters in *LRRK2* KO mice, for example serotonin in the hippocampus/amygdala may be more informative given the abnormal behaviors in the open-field.

Curiously, unlike the G2019S BAC mice, *LRRK2* KO mice do not appear to have any defect in neurogenesis, since proliferating cells and DCX counts were similar to WT mice. Dentate gyrus neurogenesis is thought to be involved in regulation of emotion [52,53] and we previously theorized that the impaired neurogenesis and anxiety phenotype may be linked in G2019S mice [26]. However, in this instance the unaltered neurogenesis in *LRRK2* KO mice rules out this idea.

Neuropathological analysis of brains from *LRRK2* KO mice does not reveal any PD-related pathology or striatal dendritic spine alterations and tau regulation also appears to be normal in KO mice. In agreement with others [34,43] we do observe a marked kidney phenotype, which in our mice is characterized by discoloration, enlargement, inflammatory and degenerative changes. The phenotype occurs in *LRRK2* KO (but not HET) from both genders and some features are observed as early as 3–4 months including discoloration, inflammation, increased p62 immunopositive cells and pigmentation. What is curious is that we observe the opposing effect on autophagy reported by Tong el al, in that we see elevated, rather than a decreased levels of LC3 II, indicating increased autophagy in the oldest (18–20 months) mice, and no indication of a biphasic response. Herzig et al recently reported that they saw no changes in LC3-II in their KO line [43], however the data presented suggests they only examined mice up to 14 months of age for this marker and we only saw quantifiable differences at the 18 month time point. Our data points toward a compensatory attempt to counteract the degeneration and pigment accumulation. Although one would expect all *LRRK2* knockout models to exhibit similar phenotypes, it is possible that subtle differences in strain background, targeting and breeding strategies may alter phenotypic progression, and perhaps if we were able to age our mice long enough, we may well observe a decrease in autophagy and alpha-synuclein accumulation in the kidney as the degenerative phenotype progresses.

## Conclusions

In summary, we report mice lacking *LRRK2* via targeted removal of the kinase domain have a normal dopaminergic system and do not develop any pathological features of PD. Our detailed behavioral analysis has revealed open-field phenotypes in KO mice, warranting further study into the role of LRRK2 and limbic system behaviors/neurochemistry. Loss of *LRRK2* has a positive impact on rotarod performance, implying possible involvement in cerebellar function and sensory processing, although the mechanisms are unclear at this time. Finally, we confirm the impact of loss of LRRK2 on the kidney, which reiterates the important consideration of the role of LRRK2 outside the CNS when designing therapeutics.

## Materials and Methods

### Animals

All animal procedures were approved by the Mayo Clinic Institutional Animal Care and Use Committee and were in accordance with the National Institute of Health Guide for the Care and Use of Laboratory Animals (NIH Publications No. 80–23) revised 1996.

### Generation of targeted LRRK2 knockout mice

*LRRK2* knockout (KO) mice, generated at Ozgene PLC (Australia) were created utilizing a construct designed to ablate *LRRK2* exon 41. Regions of 5’ homology (4 kb) and 3’ homology (4.7 kb) were used to drive the homologous recombination event by standard gene targeting techniques in C57BL/6 Bruce4 embryonic stem (ES) cells [54]. Following electroporation of the targeting construct, cells were selected for neomycin (Neo) resistance. Targeted ES cells were confirmed by Southern blotting and PCR. Euploid, targeted ES cells were then microinjected into Balb/cJ blastocysts and reimplanted into pseudopregnant dams. Resultant chimeras were bred to C57BL/6 J breeders to establish transmission. Black (i.e. those with the ES cell germline) progeny that were heterozygous for the gene-targeted allele were then bred to Cre recombinase “deleter” mice on C57BL/6 J background (Ozgene) to allow excision of the exon 41 and Neo selection cassette, which were flanked by lox P sites. Cre was then removed by breeding to C57BL/6 J wild type mice. Resultant mice were then transferred to our colony and bred to homozygosity, maintained on the C57BL/6 J background. Single nucleotide polymorphism analysis with 124 evenly spaced markers covering the mouse genome indicated that the strain was congenic on C57BL/6 with no evidence of any contaminating inbred strain.

Routine genotyping was performed by a PCR-based strategy utilizing intronic primers that span exon 41 (forward 5’CTACCAGGCTTGATGCTTTA’3, reverse 5’TCTGTGACAGGCTATATCTC’3) that yielded a 471 bp band in wild type (WT), ~220 bp band in KO and both bands in heterozygotes (HETS).

### Northern Blotting

Total RNA was extracted using Trizol® reagent (Invitrogen) according to manufacturer’s instructions. Two mice from each genotype (WT, KO, HET) were used for analysis. Total RNA (12 μg) was prepared in 1X MOPS, 6.5 % (v/v) formaldehyde and 50 % (v/v) de-ionised formamide, denatured at 65°C. Samples were electrophoresed on a denaturing gel (1 % (w/v) agarose 0.7 %, (v/v) formaldehyde, 1X MOPS, 0.005 % (v/v) ethidium bromide) for approximately 3–4 hours at 100 Volts. 0.5- 10 kb RNA ladder (Invitrogen) was used for size comparison. The RNA was then capillary transferred overnight onto Hybond-N^+^ nylon membrane (Invitrogen) and UV cross-linked. Membranes were probed with a 539 bp cDNA probe designed to exons 24–27 of mouse *LRRK2* (generated by PCR using primers forward 5’ATGCCACGTATCACCAAC’3, reverse 5’TCTAAGGTGCTGATCTGATTC’3). Probes were labeled with [α-^32^P] dCTP (3000 Ci/mmole) (Perkin Elmer) using Ready to Go labeling beads (Invitrogen). Cross-linked membranes were pre-incubated at 42°C in hybridization buffer (1X Denhardt’s solution, 4X SSC, 50 % (w/v) deionised formamide, 10 % (w/v) dextran sulphate, 200 mg/μl herring sperm DNA) for at least 30 minutes and then hybridized with labeled probe overnight at 42°C. Membranes were washed with 1X SSC for 20 minutes at room temperature to remove excess probe and then 1–2 times in 1X SSC containing 0.1 % SDS at 55°C for 15 minutes. To visualize bands, membranes were exposed to BioMax film (Kodak) at −80°C for 5–48 hours. A 214 bp *histone* cDNA probe was used as loading control (generated using primers forward 5’ GCGTGCTAGCTGGATGTCTT ‘3 and reverse 5’CCACTGAACTTCTGATTCGC ‘3).

### Antibodies

Antibody 1182E, raised to amino acids 841–960 of LRRK2 (1:200) was a gift from Dr. Benoit Giasson (Univ. Pennsylvania) was used for immunoblotting. LRRK2 immunohistochemistry was performed with MJFF2 at 1:4000 (Epitomics, c41-2) raised to amino acids 970–2527. Tyrosine hydroxylase (TH) (Affinity Bioreagents) was used to visualize dopamine neurons by immunohistochemistry (1:200) and on immunoblots (1:1000). Phospho-TH (Ser40) antibody was used for immunoblotting only (1:1000, Cell Signalling). Detection of α-synuclein was with a mouse monoclonal to α-synuclein (clone 42, 1:3500 for immunohistochemistry and 1:500 for immunoblots) from BD Transduction Labs and the phospho-Ser129 antibody (1:1000) was a gift from Dr. Takeshi Iwatsubo, University of Tokyo. Activated microglia were detected by Iba-1 (1:2000, Wako Chemicals). Tau antibodies were CP-13 (1:1000 immunohistochemistry, 1:200 immunoblots), Tau-5 (1:500 immunoblots) and PHF-1 (1:500 immunoblots) all gifts from Dr. Peter Davies, Albert Einstein College of Medicine, 12E8 (1:10,000 immunohistochemistry) a gift from Dr. Peter Seubert, Elan Pharmaceuticals and Tau-1 (1:500 immunoblots) from Millipore. For autophagy studies we used LC3 (1:500 immunoblots) from Novus and p62 (1:500 for immunoblots and 1:2000 for immunohistochemistry) from Progen. Neurogenesis studies utilized rat α-5-bromo-2-deoxyuridine (BrdU) 1:500 (Oxford Biotechnology) and goat α-doublecortin (DCX) 1:500, (Santa Cruz Biotechnology).

### Immunoblotting

Analysis of LRRK2 and tau protein was performed as previously described [47]. TH and pTH immunoblotting lysates were prepared in RIPA buffer with Triton X-100 containing protease inhibitors. 10 μg (for TH) or 50 μg (pTH) of protein was loaded onto 4-12 % Bis-Tris gels (Invitrogen). For autophagy studies samples were prepared as previously described [34], 60 μg of protein was loaded on 4-20 % Tris-glycine gels for LC3 and 10 % Tris-glycine gels for p62. ImageJ 1.42q (National Institutes of Health) was used to quantify blots. Densitometric values were analyzed statistically by either Student’s t-test or Mann Whitney non-parametric comparisons.

### Stereology

Brains from 18–20 month old KO (n = 4) and littermate WT mice (n = 4) were post-fixed in 4 % paraformaldehyde (PFA) for 24 hours followed by 30 % sucrose cryoprotection for 48 hours. Brains were sectioned exhaustively at 50 μm thickness using a freezing sledge microtome. For dopamine neuron and dendritic estimates, after a random start, every third section was stained free floating with TH antibody. Free floating immunostaining was performed utilizing the VECTASTAIN® ABC System (Vector laboratories). Sections were mounted onto glass slides, allowed to dry overnight, lightly counterstained with cresyl-violet and then dehydrated and cover slipped. Quantification was performed at high magnification (400X) using the optical fractionator number and length probes in Stereo Investigator software (MicroBrightField). Data was plotted as mean ± SEM and statistically analyzed by Student’s t-test.

### High performance liquid chromatography (HPLC)

HPLC with electrochemical detection was performed as previously described [47] in striatal tissue punches from frozen brains from mice aged 10 months KO (n = 14; 6 males, 8 females) and WT (n = 13; 7 males, 6 females) and 16–18 months KO (n = 7; 4 males, 3 females) and WT controls (n = 8; 4 males, 4 females). The amounts of monoamines/metabolites in the tissue samples were determined by comparing peak area values with those obtained from external standards run on the same day. Neurochemical concentrations were determined by normalizing samples to protein concentrations obtained from the pellets (BCA method). Data was plotted (mean ± SEM) and statistically analyzed using Mann Whitney non-parametric comparisons.

### Microdialysis

KO (n = 10 males), HET (n = 6 males) and WT littermates (n = 13 males) aged 3–4 months were anesthetized with 1-2 % isoflurane. Guide cannulae (CMA Microdialysis) were surgically implanted into the striatum using a standard stereotaxic frame (Kopf Instruments, Tujunga, CA) utilizing coordinates (from Bregma anterior-posterior +0.1 cm, lateral-medial +0.2 cm, dorso-ventral −0.2 cm) according to the Mouse Brain Atlas [55]. Mice were allowed to recover for at least 24 hours. Microdialysis experiments were carried out on conscious, freely moving mice with surgically implanted guide cannulae. On the day of the experiment, the stylet in the guide cannula was replaced with the microdialysis probe (CMA/7 with 2 mm membrane, CMA Microdialysis). The probe was perfused at 2 μl/min with artificial cerebrospinal fluid (aCSF; 145 mM NaCl, 1.2 mM CaCl_2_, 3 mM KCl, 1.0 mM MgCl_2_) for a two hour equilibration period before collection. Dialysate samples were automatically collected every 15 minutes into vials containing 2 μl perchloric acid (0.1 %) to retard oxidation of monoamines. Four baseline collections were taken at 15 minute intervals, and then the perfusate was switched to high KCl aCSF (103 mM NaCl, 1.2 mM CaCl_2_, 45 mM KCl, 1.0 mM MgCl_2_). After 30 minutes the perfusate was switched back to the original aCSF and four subsequent samples were collected every 15 minutes. Samples were analyzed by HPLC for dopamine content. Data was plotted (mean ± SEM) and statistically analyzed using Mann Whitney non-parametric comparisons.

### Pathological analysis

At least six mice from each genotype (KO, HET, WT) were analyzed per time point (3, 6, 12, 18 months). Formalin fixed, paraffin embedded tissue sections were dewaxed in xylene and rehydrated in descending alcohols and water. For antigen retrieval in paraffin sections, tissue was pressure cooked (10 minutes) in distilled water (all antibodies, except α-synuclein). Appropriate disease/tissue positive controls were included for each antibody (diffuse Lewy body disease for α-synuclein, Alzheimer for tau antibodies, Alzheimer/vascular dementia for Iba-1). Immunohistochemistry was performed using the Dako Autostainer. Tissue was quenched for endogenous peroxidases in 0.03 % H_2_O_2_ and blocked in Dako All-purpose blocking solution for 30 minutes. Primary antibody was incubated for 45 min at room temperature. All secondary antibodies were from the Envision^+^ System Labeled Polymer HRP (Dako), followed with DAB substrate (Dako), with the exception of p62 immunostaining which utilized an anti-guinea pig secondary and DAB kit (both Vector Labs). Sections were lightly counter stained in Gills 3 hematoxylin. Standard histological staining was also used (haemotoxylin and eosin, Gomori's Prussian blue, Periodic acid-Schiff and Masson Fontana).

### Transmission electron microscopy

18 month old KO and WT mice, were perfused transcardially with 2.5 % gutaraldehyde-2 % PFA in 0.1 M cacodylate buffer. Kidneys were removed, split in halves and immersed in the same fixative for two hours at room temperature. Small pieces of the cortex were further fixed in aqueous 2 % OsO_4_ and 2 % uranyl acetate, dehydrated in ethanols and propylene oxide, infiltrated and embedded in Epon 812 (Polysciences). Ultrathin sections were stained with uranyl acetate and lead citrate, and examined with a Philips 208 S electron microscope (FEI) fitted with a Gatan 831 Orius CCD camera (Gatan). Digital images were processed with Adobe Photoshop CS2 software.

### Behavioral studies

#### Open-field (OF) test

Twenty eight littermate mice (N = 8 KO males KO; 8 KO females, and N = 6 WT males; 6 WT females) were used for the evaluation of the exploratory activity in the open-field test. Open-field behavior of the mice was evaluated in a longitudinal experiment, with the first test applied at the age of 7 months and the second at the age of 16 months. Mice were habituated to the behavioral room for one week before testing. The OF apparatus consisted of a circular arena, 120 cm in diameter, surrounded by a 30 cm high wall. The apparatus, build of white plastic, was elevated 86 cm off the floor level. The arena was illuminated by 4 sets of in ceiling fluorescent lights available in a testing room and no additional illumination was used. An object (a plastic water bottle, 10 cm in diameter, 25 cm high) painted with black and white horizontal stripes was placed in the centre of the arena. All mice were individually exposed to the arena in one 5-min session. At the onset of the session, a mouse was placed near the wall of the arena and its movement on the arena throughout the duration of the session was recorded by a video tracking system (HVS Image Advanced Tracker VP200, HVS Image, Buckingham, UK). The data were extracted off-line using a Wintrack program [56].

XIn our analysis we focused on measures of motor activity in the arena and the exploration of a novel object. The following behavioral categories were used to evaluate the exploratory motor activity of mice in the open-field: *walking path length* (m) – the distance a mouse covered during the exploration of the arena, *walking speed* (m/s) – averaged speed of active walking, excluding period of rests, *latency to move* – the onset (s) of active locomotor exploration after placing a mouse in the arena, *number of stops* – a stop was defined as a period of inactivity lasting between 1 and 5 s which was separated by at least 1 s of locomotion, *number of rests* – a rest was defined as a period of inactivity lasting longer than 5 s which was separated by at least 1 s of locomotion, *time spent resting* – total time (s) spent by mice on resting, *% time in the central zone* – the percent of time spend in the central zone of the arena (50 cm radius from the centre), *thigmotaxis* – percent of time a mouse continuously walked within the close vicinity (7.5 cm) of the wall of the apparatus, *path tortuosity* (°/m) – the measure was derived by dividing the path into straight segments and curves with consistent change in direction. Following, absolute changes in direction of all curves were summed and divided by total path length. The novel object exploration was evaluated by the *latency (s) of the first approach* to the object, the *total time of object exploration* – a mouse was considered exploring an object if its nose was within a direct contact or 1 cm from an object and the body of a mouse was within a distance of 5 cm from the object perimeter, and by the *number of crosses of an object zone* – a 5 cm virtual zone surrounding an object.

A factorial model analysis of variance (ANOVA) with the genotype as between subject, and age of testing (7 and 16 months) as within subject (repeated measure) factors was used in the analysis of open-field data. While performing all repeated measures ANOVAs, departures from the assumption of compound sphericity were evaluated by Mauchly test [SPSS statistical package (SPSS Inc. Chicago) v. 19 run on a Macintosh computer] with α level set to 0.05. In cases when sphericity was significantly violated, degrees of freedom were adjusted by Greenhouse-Geisser ε-correction. Due to considerable variability of the measures obtained in the open-field [45] and relatively small sample size of mice, the interaction effect in our 2 × 2 factorial design often did not reach significance at α = 0.05. Consequently, we followed the overall ANOVA*s* by the *a priori* identified analysis focused on genotype effect at each testing age, utilizing Student’s t-tests for independent and matched-pairs samples. Correlations between the variables obtained in the open-field test were done using Pearson product–moment correlation. The critical α level for all analyses was set to 0.05. Due to space limitation, only significant results pertaining to the hypotheses testing the effect of the genotype and age are reported.

#### Rotarod

Motor co-ordination was measured using an automated rotarod system (Rotamex-5 Columbus instruments). Following a 3 day habituation period in the behavioral suite, littermate mice (7 months KO n = 9; HET n = 8 and WT n = 12) were trained for two days prior to testing. The spindle dimensions were 3.0 cm x 9.5 cm and the speed of the rod was set to 4-40 rpm acceleration, increasing 1 rpm every 5 seconds. The equipment was equipped with a sensor that automatically stops the timer if the mice cling and roll around on the rod. On the third day, mice were tested for 4 consecutive trials, allowing 10 minutes rest per trail. Data from the testing day was plotted as mean trail time and data was statistically analyzed using one way ANOVA followed by Tukey’s multiple comparisons.

#### Gait dynamics

Mice (KO n = 8; HET n = 8 and WT n = 9) were selected from the same group of animals described above for rotarod testing. Mouse gait dynamics were obtained using a motorized treadmill (with a transparent belt and digital video camera mounted underneath) by ventral plane videography [57-59] and analyzed with DigiGait® Version 9 software (Mouse Specifics, Inc). Each mouse was individually placed in the treadmill compartment for a few seconds and then the belt was turned on at a low speed (4 cm/sec) just prior to testing [previous studies show that C57BL/6 J mice do not require extended acclimatization to the treadmill [57-59]]. The motor speed was then set to 14 cm/s and at least 4 seconds of videography was collected for each mouse to obtain at least 8 sequential step images. The speed was then increased to 18 cm/s, and then 24 cm/sec, collecting an average 4 seconds of videography to obtain at least 12 or 15 sequential step images, respectively. Mice that did not have stride regularity indices (alternate step sequences) at 100 % [58,60] were still included in the study to evaluate inter-limb coordination.

Each individual gait signal per limb consists of a stance duration (time in contact with surface) and swing duration (time not in contact with surface) which together are the stride duration. Stride frequency is calculated by measuring the number of strides over time. Stride length is calculated by dividing the belt speed over the stride frequency. Paw angles and step angles at full stance are determined by software geometry calculations (fitting ellipses to the paws) of ellipse centers, major axes and vertices. The left and right gait measurements were combined for all forelimb and hindlimb data analysis. Gait indices were plotted as mean ± standard deviation and analyzed by one way ANOVA.

Supplemental methodology is also available in Additional file 7.

## Abbreviations

LRRK2/LRRK2, leucine rich repeat kinase 2; PD, Parkinson’s disease; WT, Wild type; HET, Heterozygous; KO, Knockout; BAC, Bacterial artificial chromosome; PCR, Polymerase chain reaction; TH, Tyrosine hydroxylase; DCX, Doublecortin; HPLC, High performance liquid chromatography; DA, Dopamine; DOPAC, 3,4-dihydroxyphenylacetic acid; HVA, Homovanillic acid; MSN, Medium spiny neurons.

## Competing interests

HLM, SJL and MJF have received royalties from commercial licensing of *LRRK2* KO mice. All other authors declare they have no competing interests.

## Authors’ contributions

KMH and MY performed the bulk of the husbandry and technical (molecular characterization, behavior, microdialysis surgeries and collections, HPLC, DA uptake, receptor binding, immunoblotting etc) work and contributed to manuscript writing. HLM performed microdialysis and dialysate HPLC. BB and JEB performed tissue HPLC and dendrite stereology. JCD performed immunoblotting. BB and JCD provided intellectual input to experiments and manuscript. SJL contributed intellectually to targeting design and molecular characterization. EEB performed HPLC and Golgi impregnation and analysis. CBK performed immunohistochemistry. KN performed taqman studies. IP and BW performed neurogenesis studies. CJ performed and analyzed open-field behavior and contributed to manuscript writing. WLL and DWD performed EM and pathological interpretation. HLM and MJF conceived the study. HLM designed experiments, interpreted data and wrote the manuscript. All authors read and approved the final manuscript.

## Supplementary Material

Additional file 1 **Figure S1.** No compensatory changes are observed in the expression levels of murine *LRRK1,* SNCA (*MAPT or PARKIN *genes in *LRRK2* KO mice. Real-time PCR was performed with ABI TaqMan® probes to murine (A) *LRRK1* (Mm00713303_m1), (B) murine *SNCA* (Mm00447333_m1), (C) *MAPT* (Mm00521988_m1) and (D) *PARKIN* (Mm00450187_m1). Mouse *GAPDH* (Mm99999915_m1) as the endogenous reference gene. Data plotted as mean ± SEM. In each graph/region the first column is WT, second column HET and third column is KO. Click here for file

Additional file 2 **Figure S2.** Dopamine uptake and D2 autore ceptor function is normal in LRRK2 KO mice. (A) Dopamine uptake was measured in freshly prepared synaptosomes using [2,5,6,7,8-^3^H]-DA. Each experiment included N=3 mice per genotype (8 months of age) and three independent experiments were performed. Specific DA uptake values (pmol/mg/min) were averaged and expressed as % of WT control. Data plotted as mean ± SEM. (B, C) To examine D2 autoreceptor function, mice were treated with D2 receptor antagonist raclopride and sacrificed 30 minutes later. Dopamine and dopamine metabolite levels were measured by HPLC. Dopamine turnover, defined by the ratio of (B) DOPAC/DA or (C) HVA/DA significantly increased, as expected, in all three groups (p < 0.001 ANOVA for both ratios) however post-hoc comparisons revealed the extent of this turnover increase did not differ between WT, HET and KO groups. Data plotted as mean ± SEM. Click here for file

Additional file 3 **Figure 3.** Post synaptic D1 and D2 receptor density is comparable in *LRRK2 * KO and WT mice. Quantitative autoradiography was performed with D1 receptor ligand [^3^H] SCH 23390 and D2 receptor ligand [^3^H] methylspiperone in serial striatal sections in mice aged 10 months (A,B) and 18 month (C,D). D1 and D2 binding was equivalent in KO and WT mice at both age points. Data plotted as mean ± SEM. Click here for file

Additional file 4 **Figure 4.** Loss of *LRRK2* does not impact on striatal dendritic spine density. Dendritic spines were visualized in 18 month old WT and KO mice by Golgi-Cox impregnantion and counted using Metamorph software. (A) Representative lower magnification image of a typical MSN selected for quantification - only dendrites clearly associated with an MSN-like cell body were quantified (B) High magnification of a dendrites captured by Z-stack shows that KO and WT spines appear to be comparable (C) Quantification of spines, classified by morphological type, revealed no difference between WT and KO dendrites. Data plotted as mean ± SEM. Click here for file

Additional file 5 **Figure 5.** Subgranular zone proliferation and neurogenesis are unaffected in *LRRK2* KO mice. (A) Proliferation was measured by counting BrdU positive cells in sections prepared from mice aged 4 months (N=4 per group) sacrificed 24 hours after IP BrdU injection (100mg/kg) (B) Neurogenesis was quantified in the same sections by counting doublecortin (DCX) positive neurons. Data plotted as mean ± SEM. Click here for file

Additional file 6 **Figure 6.** Tau regulation in *LRRK2* KO mice does not differ from WT. Cortical and hippocampal lysates were prepared from 18 month old WT and KO mice and immunoblots probed with tau antibodies. Graph shows representative blots for Tau-5 tau, CP-13 (pSer202) and Tau-1 in alkaline phosphatase (dephosphorylated) treated lysates. Densitometric quantification of N=6 mice per group (not shown) did not reveal any significant differences in either region for KO versus WT mice. Click here for file

Additional file 7 **Supplemental Methodology [**[26,47,61-63]**].** Click here for file
