# Supplementary figures and images for: LRRK2 knockout mice have an intact dopaminergic system but display alterations in exploratory and motor co-ordination behaviors
Source: Mol Neurodegener. 2012 May 30;7:25. doi: 10.1186/1750-1326-7-25 (PMC3441373; doi:10.1186/1750-1326-7-25)

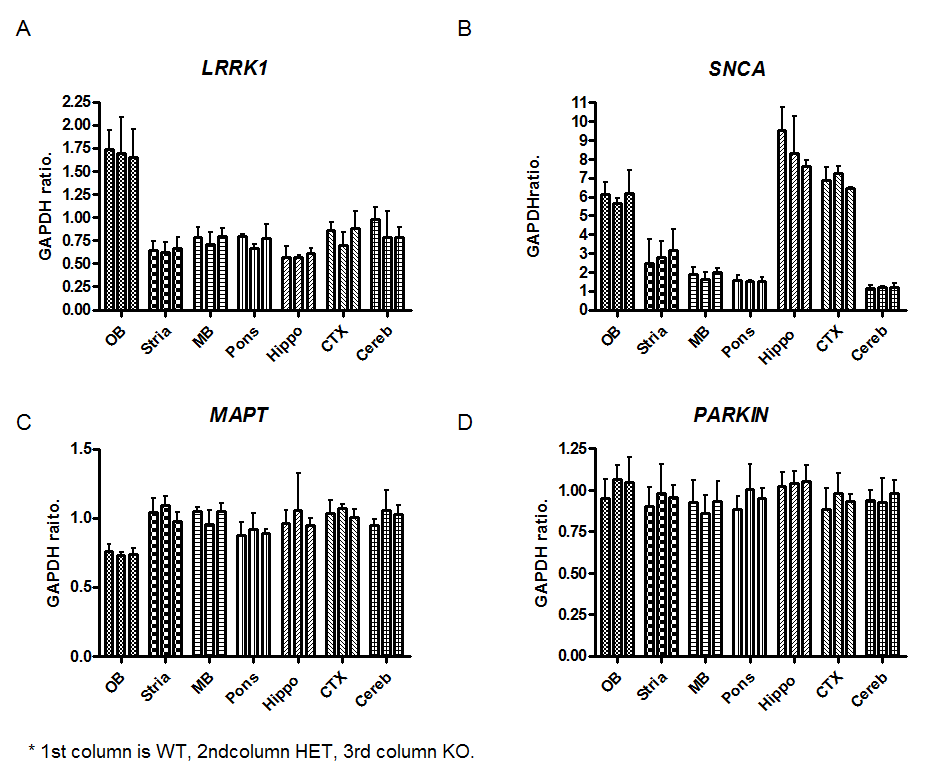

Supplement: Additional file 1 — Figure S1. No compensatory changes are observed in the expression levels of murine LRRK1, SNCA (MAPT or PARKIN genes in LRRK2 KO mice. Real-time PCR was performed with ABI TaqMan® probes to murine (A) LRRK1 (Mm00713303_m1), (B) murine SNCA (Mm00447333_m1), (C) MAPT (Mm00521988_m1) and (D) PARKIN (Mm00450187_m1). Mouse GAPDH (Mm99999915_m1) as the endogenous reference gene. Data plotted as mean ± SEM. In each graph/region the first column is WT, second column HET and third column is KO. [file 1750-1326-7-25-S1.tiff]

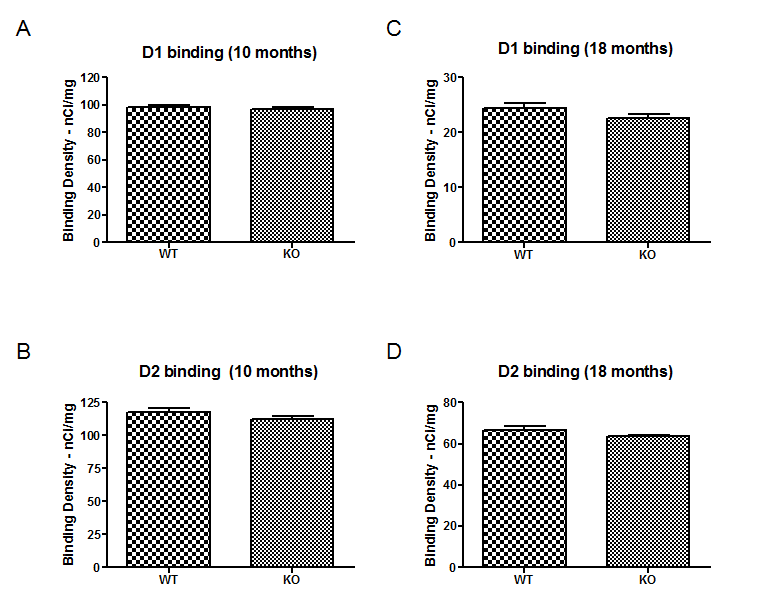

Supplement: Additional file 3 — Figure 3. Post synaptic D1 and D2 receptor density is comparable in LRRK2 KO and WT mice. Quantitative autoradiography was performed with D1 receptor ligand [3H] SCH 23390 and D2 receptor ligand [3H] methylspiperone in serial striatal sections in mice aged 10 months (A,B) and 18 month (C,D). D1 and D2 binding was equivalent in KO and WT mice at both age points. Data plotted as mean ± SEM. [file 1750-1326-7-25-S3.tiff]

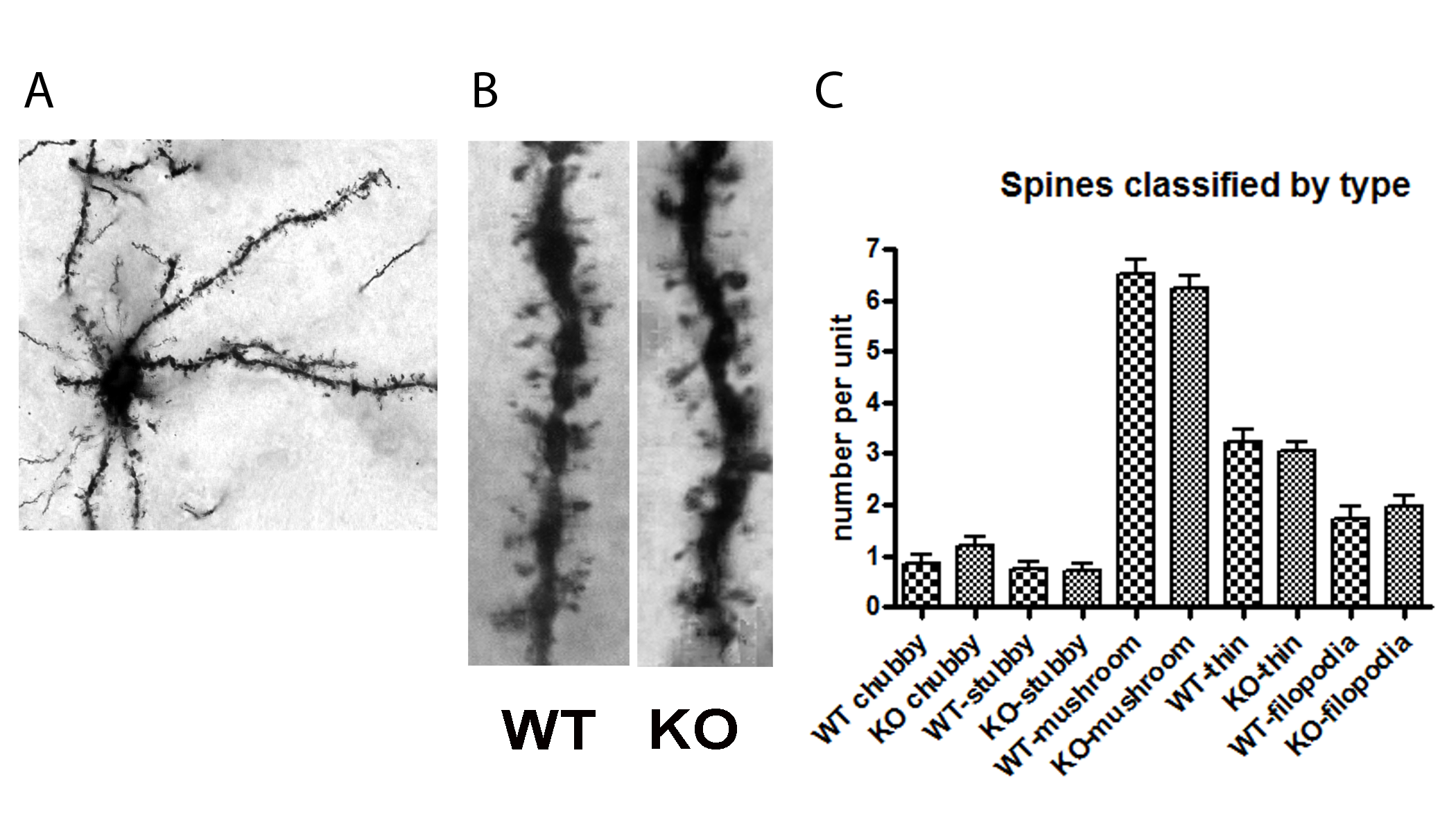

Supplement: Additional file 4 — Figure 4. Loss of LRRK2 does not impact on striatal dendritic spine density. Dendritic spines were visualized in 18 month old WT and KO mice by Golgi-Cox impregnantion and counted using Metamorph software. (A) Representative lower magnification image of a typical MSN selected for quantification - only dendrites clearly associated with an MSN-like cell body were quantified (B) High magnification of a dendrites captured by Z-stack shows that KO and WT spines appear to be comparable (C) Quantification of spines, classified by morphological type, revealed no difference between WT and KO dendrites. Data plotted as mean ± SEM. [file 1750-1326-7-25-S4.tiff]

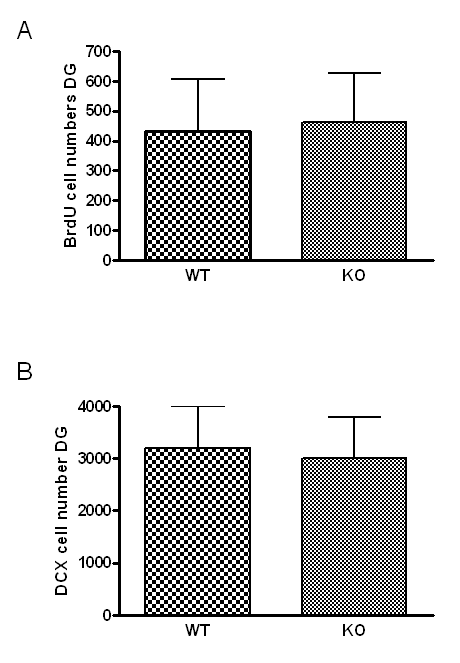

Supplement: Additional file 5 — Figure 5. Subgranular zone proliferation and neurogenesis are unaffected in LRRK2 KO mice. (A) Proliferation was measured by counting BrdU positive cells in sections prepared from mice aged 4 months (N=4 per group) sacrificed 24 hours after IP BrdU injection (100mg/kg) (B) Neurogenesis was quantified in the same sections by counting doublecortin (DCX) positive neurons. Data plotted as mean ± SEM. [file 1750-1326-7-25-S5.tiff]

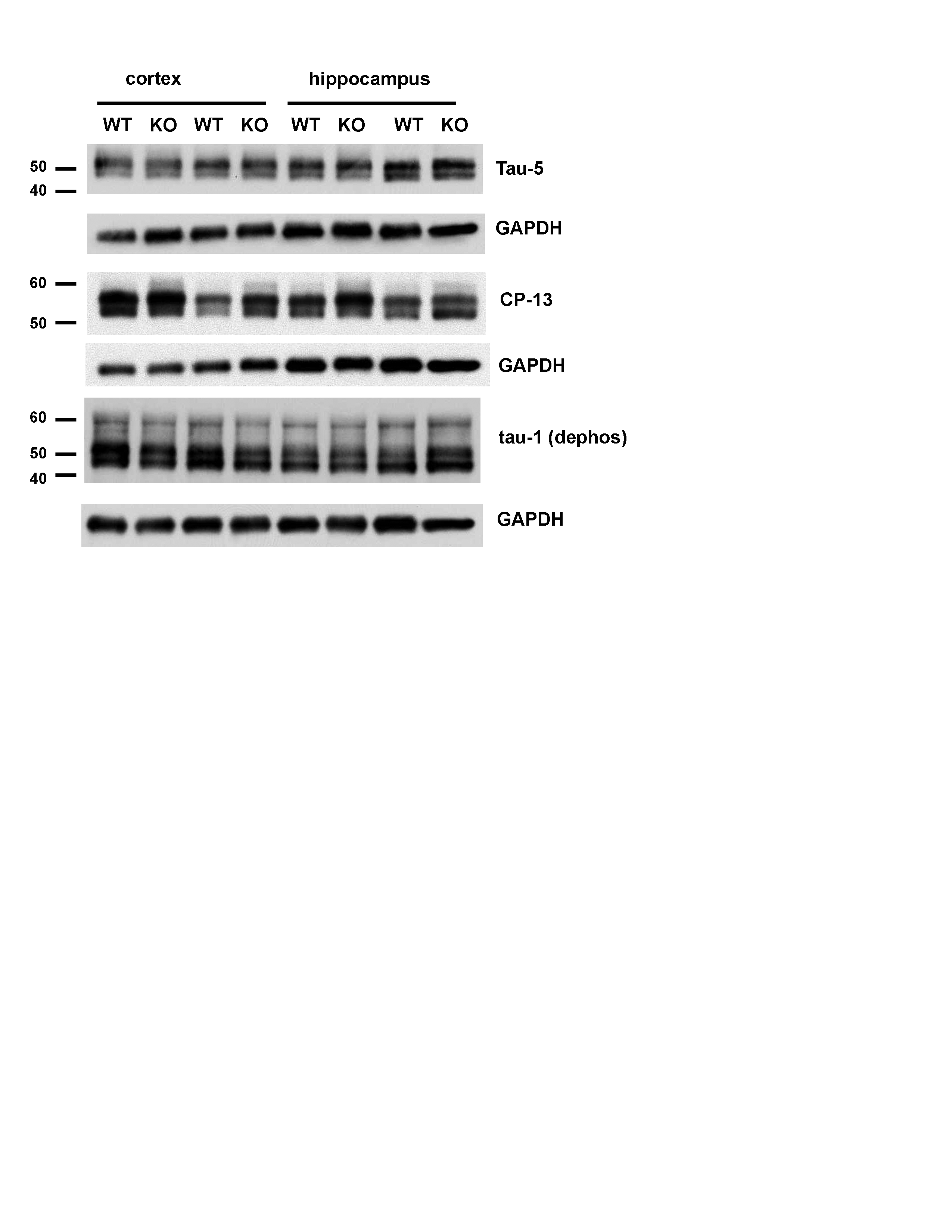

Supplement: Additional file 6 — Figure 6. Tau regulation in LRRK2 KO mice does not differ from WT. Cortical and hippocampal lysates were prepared from 18 month old WT and KO mice and immunoblots probed with tau antibodies. Graph shows representative blots for Tau-5 tau, CP-13 (pSer202) and Tau-1 in alkaline phosphatase (dephosphorylated) treated lysates. Densitometric quantification of N=6 mice per group (not shown) did not reveal any significant differences in either region for KO versus WT mice. [file 1750-1326-7-25-S6.tiff]
