# Supplementary material for: LRRK2 knockout mice have an intact dopaminergic system but display alterations in exploratory and motor co-ordination behaviors
Source: Mol Neurodegener. 2012 May 30;7:25. doi: 10.1186/1750-1326-7-25 (PMC3441373; doi:10.1186/1750-1326-7-25)
Supplement: Additional file 7 — Supplemental Methodology [[26,47,61-63]]. [file 1750-1326-7-25-S7.doc]

**Real time quantitative reverse transcriptase PCR**

Mice were sacrificed by cervical dislocation and brains were separated into different regions (olfactory bulb, hippocampus, striatum, cortex, mid-brain, brainstem, cerebellum) and frozen on dry ice. RNA was isolated using TRIzol® (Invitrogen) according to manufacturer’s instructions. cDNA was synthesized using Superscript II (Invitrogen). Real time PCR assays were performed in triplicate on a 384 well plate using an ABI 7900 detection system to assess the relative level of murine *LRRK1* (Mm00713303_ml), *SNCA* (Mm00447333_ml) and *MAPT* (Mm00521988_ml) and *PRKN* (Mm00450186_m1). In all instances murine *GAPDH* (Mm99999915_ml) was used as the endogenous reference gene.

**Dopamine Uptake Assay**

***Preparation of striatal synaptosomal fractions.***

Synaptosomes were prepared as previously described [26] with slight modifications. For each experiment N=3 mice per genotype were assayed in triplicate and the experiment was repeated three times. The mice were killed by cervical dislocation/decapitation, their brains rapidly removed and then dissected on a cooled dish. Striata were homogenized in 10 vol. of ice-cold 0.32 M sucrose/0.01M HEPES, pH 7.4 using a Potter-Elvehjem homogenizer by 10 up- and down-strokes with a Teflon pestle. The lysates were centrifuged for 10 min/1000 x g at 4°C, then the resulting crude supernatant (S1) was transferred to a new tube, discarding the pellet. The S1 lysate was centrifuged for 20 min/10,000 x g at 4°C. The resulting supernatant was discarded, and the synaptosomal pellet was resuspended in 20 vol. of ice-cold Krebs–Ringer buffer, pH 7.6 (120mM NaCl, 4.8mM KCl, 1.2mM MgSO4, 1.3mM CaCl2, KH2PO4 1.2mM, 6mM glucose, 10mM HEPES) and stored on ice. Protein was measured [61] using bovine serum albumin as a standard.

***Measurement of DA uptake in striatal synaptosomes.***

DA uptake assay was performed essentially as previously described [62] with 2uM [2,5,6,7,8-^3^H]-DA (139 Ci/mmol, Perkin Elmer). Each experiment was performed using striatal synaptosomes prepared from freshly harvested brain tissue from three mice per genotype and was repeated three times on different days. The synaptosomes in Krebs–Ringer buffer with 1uM pargyline hydrochloride were pre-incubated for 30 min at 37ºC in the presence and absence of 100uM nomifensine maleate (to determine non-specific and total DA uptake, respectively). The assay was initiated by adding 50-80ug synaptosomes (protein/assay) to [^3^H]-DA in ice-cold Krebs–Ringer buffer and incubated for 5 minutes at 37ºC. Uptake was stopped by the addition of ice-cold 0.9% saline followed by vacuum filtration (with Whatman GF/C filters presoaked in cold 0.1% polyethylenimine) using a Brandel cell harvester. The filters were rinsed 3x3mL with 0.9% saline and then transferred to scintillation vials containing 5 mL Ecoscint (National Diagnostics). Radioactive CPM values were determined by liquid scintillation spectrometry (Beckman LS6500), and specific DA uptake values (pmol/mg/min; calculated by subtracting non-specific uptake from total uptake) were averaged and per group expressed as % of the WT control.

**D2 Autoceptor analysis**

Mice aged 2 month were treated with the D2 receptor antagonist raclopride (1.0mg/kg, i.p.; Sigma) (KO n=3 WT, HET n=7, KO n= 5) or saline (WT n=3, HET n=7, KO n=4) and killed by cervical dislocation 30 minutes later. Striatal tissue punches were processed for HPLC and western blotting analysis with TH and pTH antibodies.

**D1 and D2 receptor analysis**

Dopamine D1 and D2 receptors were quantified by autoradiography with tritiated ligands as previously described (Melrose et al 2010). WT and KO mice were analyzed in striatal hemi-sections from mice aged 10 months KO (n=14; 6 males, 8 females) and WT (n=13; 7 males, 6 females) and 18 months KO (n=7; 4 males, 3 males) and WT controls (n=8; 4 males, 4 females).

**Dendritic spine analysis**

KO and WT (n=4 each) mice aged 18 months were perfused with PBS by transcardial perfusion and drop fixed in 10% buffered formalin for 24 hours. Impregnation and staining was performed using the Rapid Golgi™ Kit (FD Neurotechnologies) according to manufacturer’s instructions. Frozen brains were sectioned on a cryostat at 80μm thickness and mounted onto gelatin coated slides. Golgi impregnated striatal images were collected at 63x magnification using a Zeiss AxioImager Z1 microscope. To ensure selection of only medium spiny neurons (MSN), a dendrite was only counted when it was clearly associated with an MSN cell body. At least five neurons were imaged per section, and at least 6 sections were analyzed per mouse. For each neuron, a series of Z-stack images were collected and compressed into one single image using the extended focus module, to allow visualization of an entire dendrite. Dendritic spines were counted using Metamorph program (Molecular Devices). Counts were performed along the length of a dendrite, which was traced along in segments corresponding to 150 arbitrary units each and expressed as spines per unit.

**Neurogenesis**

Proliferation was compared in KO mice and WT littermate controls aged four months (N=4 each). Mice received a single injection of BrdU, (100 mg/kg), and were perfused with ice cold PBS, then 4% paraformaldehyde 24 hours after the injection. Brains were processed and sections stained free-floating for BrdU and doublecortin as previously described [63].

**References**

26. Winner B, Melrose HL, Zhao C, Hinkle KM, Yue M, Kent C, Braithwaite AT, Ogholikhan S, Aigner R, Winkler J, Farrer MJ, Gage FH (2011) Adult neurogenesis and neurite outgrowth are impaired in LRRK2 G2019S mice. Neurobiol Dis 41:706-716.

61. Morel P, Fauconneau B, Page G, Mirbeau T, Huguet F (1998) Inhibitory effects of ascorbic acid on dopamine uptake by rat striatal synaptosomes: relationship to lipid peroxidation and oxidation of protein sulfhydryl groups. Neurosci Res 32:171-179.

62. Bradford MM (1976) A rapid and sensitive method for the quantitation of microgram quantities of protein utilizing the principle of protein-dye binding. Anal Biochem 72:248-254.

63. Moron JA, Perez V, Fernandez-Alvarez E, Marco JL, Unzeta M (1998) "In vitro" effect of some 5-hydroxy-indolalkylamine derivatives on monoamine uptake system. J Neural Transm Suppl 52:343-349.
